# Non-stenotic Carotid Disease: Review of the Literature and Case Discussion of TCAR for Treatment

**DOI:** 10.1055/a-2893-7436

**Published:** 2026-07-24

**Authors:** Tiffany Chu, Khadija Soufi, Jose Castillo, Anzhela Moskalik, Clayton Gerndt, Branden J. Cord

**Affiliations:** 1 School of Medicine 6216 Creighton UniversityOmahaNebraskaUnited States; 2 Department of Neurosurgery 8789 University of California DavisSacramentoCaliforniaUnited States

**Keywords:** non-stenotic carotid disease, SyNC, stroke, plaque

## Abstract

Non-stenotic carotid disease represents an understudied yet clinically significant pathology. While stenosis is the primary mechanism underlying most strokes, other mechanisms such as thromboembolism from plaque rupture and carotid webs have been implicated in symptomatic non-stenotic carotid disease (SyNC). Studies have identified imaging features of high-risk non-stenotic plaques, but there is currently no standardized definition or imaging protocol for SyNC. Furthermore, despite current guidelines not recommending surgical intervention, an emerging body of literature suggests that carotid endarterectomy and carotid artery stenting may be safe and effective treatments for SyNC. In this review, we provide an overview of the existing knowledge on SyNC, including a recently proposed working definition, the underlying pathophysiology, characteristics of vulnerable plaques, and current management strategies. Additionally, we present a case from our institution using transcarotid artery revascularization (TCAR) to treat SyNC, which, to our knowledge, is the first such report in the literature.

## Introduction

Carotid artery disease has long been recognized for its significant health impacts, particularly its association with an increased risk of stroke. Strokes are most commonly classified by the Trial of Org 10172 in Acute Stroke Treatment (TOAST) system, which categorizes ischemic strokes into one of the five groups: large-artery atherosclerosis (LAA), cardioembolism, small-vessel occlusion, stroke of other determined etiology, and stroke of undetermined etiology. 1 Management of carotid artery disease has been well-studied with landmark trials such as the North American Symptomatic Carotid Endarterectomy Trial (NASCET) and European Carotid Surgery Trial (ECST), among others. 2 3 These trials have been instrumental in shaping our current approach to managing this disease, which is largely based on the severity of luminal stenosis being greater than 70%. 4 5

With advances in imaging methods over the years, it has become easier to identify carotid artery plaques. With this, so has the identification of non-stenotic carotid plaques in patients presenting with stroke-like symptoms. Studies have emerged highlighting symptomatic non-stenotic carotid disease (SyNC) as a common cause of strokes as well. Most commonly characterized as having less than 50% luminal stenosis, SyNC is considered an embolic stroke of undetermined source (ESUS) under the TOAST classification system. Although SyNC is a diagnosis of exclusion, it has been reported to account for up to 25% of ischemic cerebrovascular events. 6 Studies utilizing data from the ESCAPE-NA1 trial, INTERRSeCT trial, and STRATIS registry have even identified an association between SyNC and ipsilateral ischemic stroke. 7 8 9

Although the overall stroke risk in non-stenotic carotid plaques is on average lower than that in stenotic plaques, the risk of stroke is not irrelevant. As a result, it is important to understand the high-risk features that could potentially increase the risk of arterio-arterial embolic stroke as well as the options providers have when dealing with this pathology. In this article, we provide an overview of epidemiological, clinical, and imaging features of SyNC, and summarize the scientific evidence and current practice patterns with regard to SyNC treatment strategies. Along with our review of current studies describing SyNC, we have included case examples of different presentations and treatments for SyNC in two patients from our institution.

## SyNC Definition

Literature has varied significantly in terms of defined thresholds for SyNC, with numerous studies analyzing patient cohorts with <70% luminal stenosis, most commonly <50% luminal stenosis, and one study using a lower threshold of <30% luminal stenosis. 10 Beyond focusing on luminal stenosis, some studies have also attempted to characterize SyNC with morphological plaque features (plaque ulceration, intraplaque hemorrhage, plaque thickness >3 mm) or imaging findings consistent with recurrent embolic stroke confined to a corresponding internal carotid artery (ICA) territory. 11 12

Despite the absence of a formally standardized definition for SyNC, a working definition has been proposed by Goyal et al in recent years. 13 This definition accounts for imaging findings and the likelihood of other stroke etiologies, ultimately leading to a classification system that stratifies plaques into possible, probable, or definite SyNC. In summary, they ultimately conclude the definition requires the presence of a non-stenotic (<50%) carotid plaque and imaging findings consistent with acute embolic stroke(s) confined to the corresponding ICA territory.

## Epidemiology

Among patients with ESUS, the prevalence of non-stenotic carotid disease has been reported to range from 22.7 to 39.1%. 7 8 9 Notably, patients with SyNC are generally younger compared with those suffering strokes from other etiologies. 6 It has been well-established that the degree of stenosis increases the risk of stroke, but patients with non-stenotic carotid disease do not have a negligible risk of stroke. 14 The incidence of stroke or transient ischemic attack (TIA) in this population has been reported to be 0.5 per 100 person-years, and this risk increases with certain patient factors and plaque characteristics. 15 For instance, patients with a prior history of stroke have an annual recurrence rate of 2.6%. 15 Additionally, the presence of high-risk features of the plaque, such as thickness, ulceration, and intraplaque hemorrhage, further elevates this risk. 13 15 Future studies are needed to better understand the epidemiology of this disease.

## Pathophysiology

### Plaque Formation and Progression

The mechanisms driving the formation and progression of plaques in non-stenotic carotid disease are similar to those of stenotic carotid artery disease. In this multifactorial disease, various factors such as hypertension, smoking, and genetic susceptibility contribute to the development of atherosclerosis. 16 Endothelial dysfunction leads to the accumulation of low-density lipoprotein (LDL) cholesterol within the arterial wall, triggering an inflammatory response which activates vascular smooth muscle cells. 17 These smooth muscle cells, as well as the collagen and proteoglycans that they produce, form the fibrous cap of the plaque. 18 As the intima progressively thickens, the metabolic demands of the tissue exceed the progressively worsening oxygen diffusion. 19 This, coupled with the activation of inflammation-driven pathways, causes new vessels to form within the plaque. Although the neovascularization process is crucial for maintaining plaque stability which prevents intraplaque hemorrhage, studies have also demonstrated that increased neovascularization is associated with a higher risk of ischemic events. 20

### Underlying Mechanisms of Stroke

The classically understood mechanism of how stenosis can lead to strokes proposes that a decrease in blood flow leads to hypoperfusion and ischemia. 21 22 However, some studies have proposed alternative mechanisms—thromboembolism from plaque rupture or acute occlusion of the carotid artery with subsequent propagation of the thrombus into arteries supplying the brain. 21 Research has shown that higher wall shear stress proximal to the region with the most severe stenosis reduces proliferation, which causes thinning of the fibrous cap and plaque instability, predisposing it to rupture. 23 24 25 Additionally, studies demonstrating that most strokes do not occur in watershed areas provide support against the proposed ischemic pathophysiologic mechanism. 26 These mechanisms highlight the multifaceted interaction between fluid hemodynamics and plaque composition in contributing to the risk of stroke in patients with non-stenotic carotid disease. Both of these reasons remain as explanations behind SyNC patients becoming symptomatic, with the latter believed to be most relevant.

### Carotid Webs

In addition to atherosclerotic plaques, carotid webs are another important mechanism contributing to non-stenotic carotid disease. This rare form of fibromuscular dysplasia is characterized by the presence of fibrous non-atherosclerotic tissue arising from the posterior wall of the internal carotid bulb and projecting into the arterial lumen. 27 Despite causing minimal stenosis, typically ranging from 0 to 20% based on NASCET criteria, carotid webs have been recognized as risk factors for ischemic strokes. 28 29 30 31 32 Strokes of this etiology are particularly common in patients with few or no traditional risk factors, with a systematic review identifying only 43% of their cohort as having at least one conventional risk factor for ischemic stroke. 33 Studies have also found that patients affected by these strokes tended to be younger (median age of 46 years), female, and of African descent. 34 It is thought that the disruption of laminar flow distal to the carotid web contributes to thrombus formation and subsequent embolic strokes. 30 35 This pathophysiologic mechanism is supported by recent studies demonstrating that patients with symptomatic carotid webs who underwent carotid revascularization had a 0% rate of stroke recurrence, compared with a rate of 26 to 56% in those managed medically. 33 36 We argue carotid webs should be considered as a subcategory of SyNC, even though they are not always associated with plaque. Future studies are needed to assess the relationship of carotid webs to SyNC.

## Assessing Plaque Characteristics

Identifying high-risk plaque features is crucial to determining stroke risk and guiding management. Intraplaque hemorrhage, lipid-rich necrotic cores, thin or ruptured fibrous caps, plaque ulceration, and neovascularization are key indicators of embolic potential ( Table 1 ). 37

**Table 1:** High-risk features of non-stenotic plaques with associated imaging modalities

Feature	Imaging modality for detection
Intraplaque hemorrhage	MRI
Lipid-rich necrotic core	MRI, CTA
Thin or ruptured fibrous cap	MRI
Ulceration	MRI, CTA
Hypoechogenicity	B-mode ultrasonography
Microembolic signals	CEUS
Neovascularization	CEUS

Abbreviations: CEUS, contrast-enhanced ultrasound; CTA, computed tomography angiography; MRI, magnetic resonance imaging.

### Magnetic Resonance Imaging

Magnetic resonance imaging (MRI) is one of the most sensitive modalities for detecting high-risk plaque features. Black-blood MRI sequences, such as T1-weighted and magnetization-prepared rapid acquisition gradient-echo (MPRAGE), highlight hemorrhagic components within the plaque, which are a major predictor of stroke risk. 38 In particular, as the most common feature of complicated plaques, intraplaque hemorrhage (IPH) has been shown to be most strongly correlated with risk of recurrent ischemic stroke. 39 MRI also enables assessment of lipid-rich necrotic cores (LRNCs), plaque ulceration, and fibrous cap integrity, which are associated with increased stroke risk. 40 41 42

### Computed Tomography Angiography

Computed tomography angiography (CTA) is a widely available and fast imaging modality that can evaluate plaque morphology and luminal stenosis. High-risk CTA features include plaque ulceration, low-attenuation plaques (indicative of lipid-rich content or intraplaque hemorrhage), and plaque irregularity. 34 43 Although CTA is less sensitive than MRI in detecting intraplaque hemorrhage, it remains a valuable first-line imaging tool in the acute setting due to its accessibility and rapid acquisition. 12 44 45

### Ultrasonography

Carotid ultrasound is a valuable non-invasive tool for assessing plaque characteristics. The primary techniques utilized are B-mode ultrasonography, transcranial doppler ultrasonography (TCD), and contrast-enhanced ultrasound (CEUS). 46 B-mode ultrasonography evaluates plaque echogenicity, TCD identifies the presence of microembolic signals, and CEUS assesses plaque morphology and neovascularization.

In B-mode ultrasonography, ultrasound waves generate echoes as they encounter irregularities of the tissue, such as arterial plaques. The echogenicity of plaques correlates with their composition and can be classified into hypoechoic, isoechoic, or hyperechoic. Hypoechoic plaques are suggestive of lipid-rich content and associated with hemorrhage, both features of vulnerable plaques. 47 48 These hypoechoic plaques are more commonly observed in symptomatic plaques compared with asymptomatic ones. 49 Moreover, the presence of hypoechoic plaques has been correlated with an increased risk of stroke, independent of the degree of stenosis. 50 51

The findings from B-mode ultrasonography can be used in conjunction with transcranial doppler ultrasonography (TCD) to further stratify the risk of stroke. TCD can detect the presence of microemboli in the intracranial circulation, which supports the embolic potential of carotid plaques. 52 These microembolic signals (MES) have been demonstrated to be markers of vulnerable plaque, with TCD detecting MES in 43% of symptomatic patients with carotid stenosis, compared with only 10% in asymptomatic patients. 53 The presence of MES has also been correlated with an increased likelihood of future stroke events. Meanwhile, a prospective study by Spence et al found that patients without these signals had less than a 1% risk of stroke at 1-year follow-up. 54 Furthermore, the presence of both hypoechogenicity on B-mode ultrasonography and MES on TCD in patients with asymptomatic carotid stenosis has been linked with a 10-fold greater risk of stroke compared with those without both findings. This association remained significant even after accounting for the degree of stenosis. 55

As opposed to the contrast utilized in CT and MRI, CEUS employs microbubble contrast agents which remain intravascular. 56 57 This coupled with the spatial and temporal resolution of CEUS allow for enhanced differentiation of plaque components as well as real-time observation of blood flow and diffusion patterns of the contrast. These features make CEUS particularly well-suited for identifying and quantitatively grading plaque neovascularization, a marker of plaque vulnerability. 58 59 Several grading systems have been published, ranging from two- to five-level systems. 38 60 61 62 63 64 These grading systems categorize neovascularization based on the extent and location of contrast enhancement, with higher grades reflecting greater neovascularization. 63 Importantly, neovascularization identified with CEUS has been demonstrated to be an independent predictor of stroke recurrence in patients with anterior circulation ischemic strokes and atherosclerosis. 65

### Positron Emission Tomography

Positron emission tomography (PET)/CT and PET/MRI provide novel insights into plaque biology by detecting metabolic activity associated with inflammation. Increased FDG uptake correlates with macrophage activity and inflammation, both of which are associated with plaque instability. Novel PET tracers targeting specific components of vulnerable plaques, such as fibrin or matrix metalloproteinases, are being investigated to further refine risk stratification. 45 66

## Medical and Lifestyle Management

Given the classification of SyNC as ESUS, there currently do not exist any specific guidelines for managing SyNC. General practice focuses on managing risk factors that increase the risk of thromboembolic events. This typically includes systemic anticoagulation or dual antiplatelet therapy (DAPT) to minimize plaque erosion. Although anticoagulation is traditionally thought to more directly impact the pathway implicated in cardioembolic strokes, three large-scale international randomized controlled trials, NAVIGATE, RE-SPECT ESUS, and ARCADIA, each with over 3,500 patients, found no superiority of rivaroxaban, dabigatran, or apixaban over aspirin in preventing recurrent embolic strokes. 67 Notably, the NAVIGATE trial was terminated after 11 months due to the lack of benefit identified in the rivaroxaban cohort (5.1% annual risk of stroke recurrence or systemic embolism versus 4.8% in the aspirin cohort) as well as the increased rate of major bleeding (1.8 vs. 0.7%, *p* < 0.001). 68 Similarly, the RE-SPECT ESUS trial found that dabigatran conferred no significant difference in annual risk of stroke recurrence compared with aspirin (4.1 vs. 4.8%), nor in major bleeding risk (1.7 vs. 1.4%). 69 In the ARCADIA trial, which studied patients with atrial cardiopathy without atrial fibrillation, study authors identified no difference in stroke recurrence (4.4 vs. 4.4%), intracranial hemorrhage (0 vs. 1.1%), or other major hemorrhage (0.7 vs. 0.8%) between the apixaban and aspirin cohorts. 70

Additional management strategies include controlling blood pressure and cholesterol levels with antihypertensives and statins, respectively. 44 71 Combination therapy with angiotensin-converting enzyme (ACE) inhibitors and statins has been shown to have synergistic effects in reducing plaque volume and stabilizing plaque. 72 Lifestyle modifications such as healthy diet, regular exercise, weight loss, and smoking cessation are also essential to reducing overall cardiovascular risk. 73

## Surgical Management

Since current guidelines for the treatment of carotid strokes are primarily based on the degree of stenosis, surgical management of SyNC is not supported. Both the Society for Vascular Surgery and European Society for Vascular Surgery (ESVS) do not recommend carotid endarterectomy (CEA) in patients with less than 50% stenosis. However, ESVS does note that CEA or carotid artery stenting (CAS) could be considered for patients with recurrent embolic events despite maximal medical therapy. 4 5 Nonetheless, some studies have evaluated the safety and efficacy of CEA and CAS for managing SyNC.

### Carotid Endarterectomy

In a single-center retrospective case series, Nardi et al evaluated 32 patients with SyNC who underwent CEA for secondary prevention of stroke. They reported no intraoperative complications or ischemic events at last follow-up. 74 Similarly, a prospective cohort study found CEA to be an effective procedure in reducing the risk of recurrent stroke in patients with SyNC, particularly those with high-risk plaques, such as those with lipid-rich necrotic cores and intraplaque hemorrhage. 75 It is important to note that most of these studies involved patients who were receiving medical therapy—either DAPT or at least one antiplatelet agent—prior to the CEA. 74 75 76 77 78 79 As a procedure that carries a higher risk profile compared with CES or TCAR, its application has been limited for SyNC especially in the context of guidelines. 80

### Carotid Artery Stenting

For patients with high surgical risk profiles, CAS has emerged as an alternative to CEA. Despite the absence of guideline support, several studies have explored CAS utility in managing SyNC. A prospective single-institutional study by de Haro et al demonstrated that CAS was both safe and effective in preventing recurrent strokes in patients with symptomatic low-grade stenosis. Their cohort reported no perioperative complications or ischemic events during the follow-up period, which averaged 30.3 months. 81 Beyond stroke risk reduction, a small prospective study has shown that both CEA and CAS performed after the initial ischemic event effectively prevented subsequent restenosis or stroke. 79

From a procedural perspective, contemporary carotid stents were designed primarily for stenotic vessels. Their substantial radial force can result in vessel wall trauma, while their relatively large pore dimensions permit passage of plaque debris into the lumen, potentially increasing periprocedural embolic complications. Furthermore, the comprehensive metal coverage renders subsequent CEA technically unfeasible due to the surgical inability to incise through the dense wire infrastructure. However, the absence of robust data limits conclusions regarding the superiority of CEA, CAS, or aggressive medical management in treating non-stenotic carotid artery disease. Future studies are needed to better answer this question.

### Transcarotid Artery Revascularization

Transcarotid artery revascularization (TCAR) is a carotid stenting method that avoids the manipulation of the aortic arch as one obtains access via direct puncture of the surgically exposed common carotid artery. TCAR also uses a flow-reversal neuroprotection system, reducing the risk of embolic events which is of particular importance for those with high-risk plaques. To our knowledge it has never been described in the literature for treating a patient with SyNC. Anecdotally, we argue it should be a highly considered option when compared with CEA or traditional CAS as studies have shown a lower risk of stroke or death compared with the transfemoral CAS approach. 82 Even though it has been shown to have an equivalent risk of stroke or death compared with CEA, TCAR does have a lower risk of myocardial infarction and cranial nerve injuries. In the hands of an experienced surgeon, it has been shown to lead to shorter operative times as well. Below, we describe a case of TCAR being used to treat a patient with SyNC at our institution.

#### Case

**Fig. 1:**
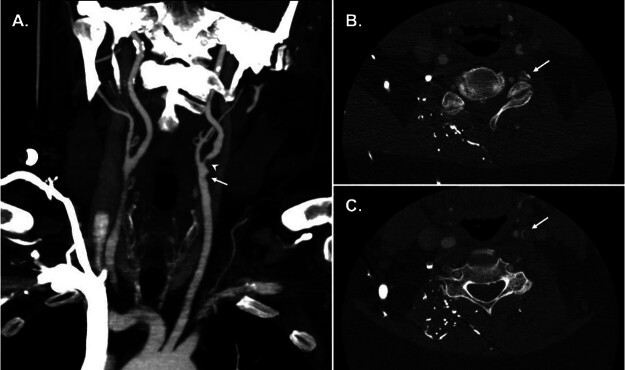
CT angiogram of the neck demonstrating ( **A** ) coronal view of left common carotid (arrow) and ICA (arrowhead) stenosis, ( **B** ) axial view of left common carotid stenosis, and ( **C** ) axial view of left ICA stenosis. CT, computed tomography; ICA, internal carotid artery.

**Fig. 2:**
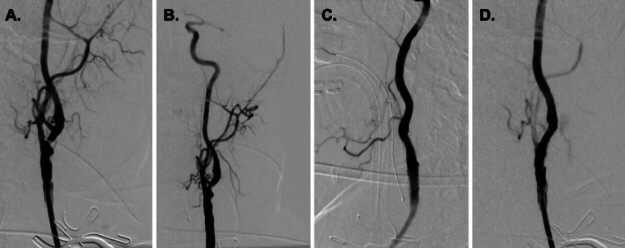
Digital subtraction angiography of carotid stenosis before transcarotid artery revascularization ( **A, B** ) and after ( **C, D** ).

A 72-year-old man with history of bilateral CEA, atrial fibrillation, hypertension, and multiple strokes initially presented with new-onset intermittent blurriness in the left eye. He was found to have a left ICA tethered thrombus and was discharged on apixaban and clopidogrel. The patient returned 2 weeks later with worsening visual symptoms that progressed to loss of vision in the left eye. Repeat imaging demonstrated persistence of the tethered thrombus ( Fig. 1 ). Due to the history of prior CEA resulting in scar tissue and a more difficult neck dissection, and concern for embolism of the tethered thrombus during surgical manipulation of the vessel, TCAR was preferred since flow reversal could be established prior to any manipulation of the diseased segment ( Fig. 2 ). Postoperatively, the patient reported that his symptoms returned to baseline.

## Conclusion

SyNC remains an under-reported and under-recognized source of strokes, partly due to its current categorization within the TOAST classification system. As a stroke of undetermined etiology, few guidelines exist regarding the diagnosis and management of this condition. The limited studies that have been reported on SyNC utilize varying definitions, which further complicates our understanding of its prevalence, risk factors, and management. Additional research, including randomized controlled trials with long-term follow-up, is necessary to better understand the implications of this disease. Given the high risk of recurrent stroke in this population, these studies will be crucial in developing more standardized recommendations and guidelines.
